# Restricted Sequence Variation in Streptococcus pyogenes Penicillin Binding Proteins

**DOI:** 10.1128/mSphere.00090-20

**Published:** 2020-04-29

**Authors:** Andrew Hayes, Jake A. Lacey, Jacqueline M. Morris, Mark R. Davies, Steven Y. C. Tong

**Affiliations:** a Department of Microbiology and Immunology, University of Melbourne, Peter Doherty Institute for Infection and Immunity, Melbourne, Victoria, Australia; b Doherty Department, The Peter Doherty Institute for Infection and Immunity, The University of Melbourne and The Royal Melbourne Hospital, Melbourne, Victoria, Australia; c Victorian Infectious Disease Service, The Royal Melbourne Hospital, Peter Doherty Institute for Infection and Immunity, Melbourne, Victoria, Australia; d Menzies School of Health Research, Charles Darwin University, Darwin, Northern Territory, Australia; U.S. Centers for Disease Control and Prevention

**Keywords:** *Streptococcus pyogenes*, beta-lactams, penicillin resistance, penicillin binding proteins

## Abstract

A recent clinical report has linked Streptococcus pyogenes β-lactam antibiotic resistance to mutation in the penicillin binding protein (PBP) PBP2x. To determine whether this is an isolated case or reflects a broader prevalence of mutations that might confer reduced β-lactam susceptibility, we investigated the relative frequency of PBP sequence variation within a global database of 9,667 S. pyogenes isolates. We found that mutations in S. pyogenes PBPs (PBP2x, PBP1a, PBP1b, and PBP2a) occur infrequently across this global database, with fewer than 3 amino acid changes differing between >99% of the global population. Only 4 of the 9,667 strains contained mutations near transpeptidase active sites of PBP2x or PBP1a. The reported PBP2x T553K substitution was not identified. These findings are in contrast to those of 2,520 *S. pneumococcus* sequences where PBP mutations are relatively frequent and are often located in key β-lactam binding pockets. These data, combined with the general lack of penicillin resistance reported in S. pyogenes worldwide, suggests that extensive, unknown constraints restrict S. pyogenes PBP sequence plasticity. Our findings imply that while heavy antibiotic pressure may select for mutations in the PBPs, there is currently no evidence of such mutations becoming fixed in the S. pyogenes population or that mutations are being sequentially acquired in the PBPs.

**IMPORTANCE** β-Lactam antibiotics are the first-line therapeutic option for Streptococcus pyogenes infections. Despite the global high prevalence of S. pyogenes infections and widespread use of β-lactams worldwide, reports of resistance to β-lactam antibiotics, such as penicillin, have been incredibly rare. Recently, β-lactam resistance, as defined by clinical breakpoints, was detected in two clinical S. pyogenes isolates with accompanying mutations in the active site of the penicillin binding protein PBP2x, raising concerns that β-lactam resistance will become more widespread. We screened a global database of S. pyogenes genome sequences to investigate the frequency of PBP mutations, identifying that PBP mutations are uncommon relative to those of Streptococcus pneumoniae. These findings support clinical observations that β-lactam resistance is rare in S. pyogenes and suggest that there are considerable constraints on S. pyogenes PBP sequence variation.

## Abstract (precis)

β-Lactam antibiotics are the first-line therapeutic option for Streptococcus pyogenes infections. Despite the global high prevalence of S. pyogenes infections and widespread use of β-lactams worldwide, reports of resistance to β-lactam antibiotics, such as penicillin, have been incredibly rare. Recently, β-lactam resistance, as defined by clinical breakpoints, was detected in two clinical S. pyogenes isolates with accompanying mutations in the active site of the penicillin binding protein PBP2x, raising concerns that β-lactam resistance will become more widespread. We screened a global database of S. pyogenes genome sequences to investigate the frequency of PBP mutations, identifying that PBP mutations are uncommon relative to those of Streptococcus pneumoniae. These findings support clinical observations that β-lactam resistance is rare in S. pyogenes and suggest that there are considerable constraints on S. pyogenes PBP sequence variation.

## INTRODUCTION

Streptococcus pyogenes (group A *Streptococcus*, or GAS) has previously been understood to be uniformly susceptible to β-lactam antibiotics (1). Two S. pyogenes isolates with elevated MICs to β-lactam antibiotics have recently been reported (2). Both isolates were molecularly typed as *emm*43.4 and had a penicillin binding protein (PBP) PBP2x missense mutation (T553K) at the transpeptidase active site, which was associated with an 8-fold and 3-fold increased MIC to ampicillin and cefotaxime, respectively, compared to levels for closely related isolates without the PBP2x mutation. In contrast to S. pyogenes, reduced susceptibility to β-lactams has been widely reported in S. pneumoniae and is strongly associated with sequence variation in PBPs (3, 4).

Using GAS genome sequences from global sources, we sought to determine the prevalence of substitutions across the transpeptidase domains of the GAS PBPs (PBP2x, PBP1a, PBP2a, and PBP1b) compared with domains of S. pneumoniae (which shares PBP2x and PBP1a).

## RESULTS

We examined sequence variation in PBP1a, PBP1b, PBP2a, and PBP2x among 9,667 S. pyogenes genome sequences, representing 115 different *emm* types and 321 multilocus sequence types (see Table S1 and Text S1 to S^4^ in the supplemental material). These genome sequences were mostly from data sets from the United Kingdom and United States that focused on invasive disease (5–16). Mutations in the penicillin binding proteins (PBPs) have been associated with reduced clinical β-lactam susceptibility for S. pneumoniae (4), S. agalactiae (17), S. dysgalactiae (18), and now S. pyogenes (2). A comparison of PBP2x between β-lactam-susceptible reference genomes of S. pyogenes, S. pneumoniae, S. agalactiae, and S. dysgalactiae subspecies *equisimilis* demonstrated a high level of interspecies conservation (>72% similarity) (Fig. S1 and Table S2). In S. pneumoniae, substitutions at the PBP2x transpeptidase active site (SXXK, SXN, and KSTG) result in reduced β-lactam susceptibility. These three motifs were conserved across the four species (Fig. S1).

10.1128/mSphere.00090-20.1 FIG S1 PBP2x alignment for S. pyogenes, S. pneumoniae, S. agalactiae, and S. dysgalactiae subspecies *equisimilis.* Clustal Omega multiple-sequence alignment of PBP2x among S. pyogenes serotype M3 strain ATCC BAA-595/MGAS315 (genome reference NC_004070.1, protein reference WP_011106648.1), S. pneumoniae strain ATCC BAA-255/R6 (genome reference NC_003098.1, protein reference WP_000872267.1), S. agalactiae strain 2603V/R (genome reference NC_004116.1, protein reference WP_000142542.1), and the S. dysgalactiae subspecies *equisimilis* (labelled *S. equisimilis*) strain RE378 (genome reference NC_018712.1, protein reference WP_015017311.1). The three transpeptidase active-site motifs are highlighted in boldface and underlined. The residue highlighted in red (T553) refers to the substitution associated with clinical β-lactam resistance in S. pyogenes (2). Fully conserved amino acids are denoted by an asterisk, a strongly conserved protein is denoted by a colon, and proteins that are weakly conserved are denoted by a full stop. Download FIG S1, PDF file, 0.05 MB. Copyright © 2020 Hayes et al. 2020 Hayes et al. This content is distributed under the terms of the Creative Commons Attribution 4.0 International license.

10.1128/mSphere.00090-20.4 TABLE S1 Strain list with *emm* and MLST types, amino acid sequence, and alleles of PBP2x, PBP1a, PBP1b, and PBP2a (see Text S1 to S4). Download Table S1, XLSX file, 0.4 MB. Copyright © 2020 Hayes et al. 2020 Hayes et al. This content is distributed under the terms of the Creative Commons Attribution 4.0 International license.

10.1128/mSphere.00090-20.5 TABLE S2 Similarity matrix between PBP2x for four *Streptococcus* species, as determined by BLOSUM62 threshold of ≥1. Download Table S2, DOCX file, 0.02 MB. Copyright © 2020 Hayes et al. 2020 Hayes et al. This content is distributed under the terms of the Creative Commons Attribution 4.0 International license.

**FIG 1:**
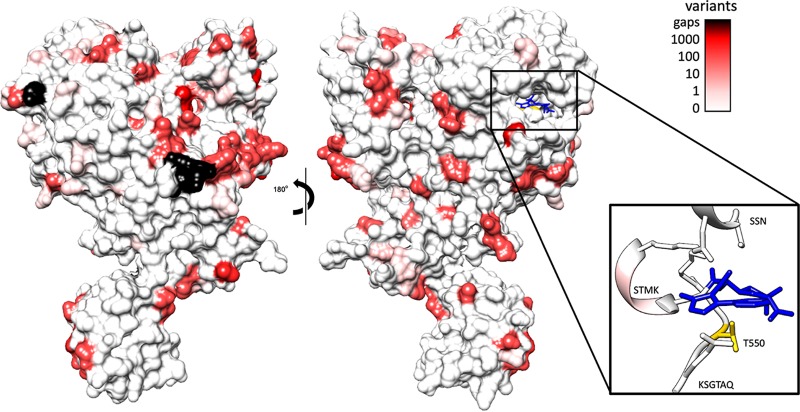
Global amino acid variation of Streptococcus pyogenes PBP2x mapped against the crystal structure of Streptococcus pneumoniae PBP2x. Crystal structure of PBP2x from S. pneumoniae (PDB entry 5OIZ) bound to oxacillin (blue), with the frequency of residue conservation from 9,667 S. pyogenes PBP2x sequences represented as a color gradient. Black residues represent regions absent from the alignment due to the absence of sequence relative to the S. pneumoniae crystal structure. Thresholds were chosen to represent differing orders of magnitude for conservation, with thresholds set at orders of magnitude (0, 1, 10, 100, and 1,000 sequences varying at the residue). (Inset) Ribbon diagram of binding pocket motifs SSN, STMK, and KSG with the position of the mutated residue (T553K) highlighted (yellow). Mutations were observed in the STMK motif in 4 of the 9,667 sequences.

Given the similarity between PBP2x of S. pneumoniae and S. pyogenes (73.4% similarity) (Table S1), we mapped the conservation of residues from the alignment of 9,667 S. pyogenes PBP2x sequences onto the crystal structure of S. pneumoniae PBP2x (Fig. 1). The transpeptidase active-site motifs are SXXK at positions 340 to 343 in S. pyogenes (positions 337 to 340 in S. pneumoniae), SXN at positions 399 to 401 in S. pyogenes (positions 395 to 397 in S. pneumoniae), and KSGT at positions 550 to 553 in S. pyogenes (positions 547 to 550 in S. pneumoniae). There were 101 unique amino acid sequence variants of the S. pyogenes PBP2x sequence, with no frameshifts or premature stop codons (Text S1 and Table S3). We found no instances of the T553K substitution in the PBP2x KSGT motif, as reported in the recent S. pyogenes β-lactam-resistant isolates (2). Only four S. pyogenes isolate sequences (0.04%) had substitutions within the transpeptidase active-site motifs of PBP2x (Fig. 1 and Table 1), corresponding to STMK to SAMK and STMK to STIK. These changes may not have a phenotypic effect on penicillin susceptibility, as STIK recently has been reported in a penicillin-susceptible isolate (GASAR0057) (16). Another 84 (0.9%) of the 9,667 genomes contained mutations at one of four amino acid positions associated with increased tolerance to subclinical β-lactam MIC identified through a recent population genomics study of *emm*1, *emm*28, and *emm*89 S. pyogenes (Table S3) (19). Furthermore, no amino acid substitutions were found in the active-site motifs of S. pyogenes PBP1a. In comparison, using population data from Li et al. (4), S. pneumoniae had active-site motif variants in 639/2,520 (25.3%) isolates for PBP2x and 445/2,520 (17.7%) for PBP1a (Table 1). A large proportion of S. pneumoniae substitutions mapped to areas near the active site (Fig. S2).

**TABLE 1:** Percentage of transpeptidase sequences with variation in the SXXK, SXN, or K(T/S)G motif of the transpeptidase active sites in PBP1a and PBP2x for S. pneumoniae and S. pyogenes

Motif	No. (%) of variants in:	
	S. pneumoniae (*n* = 2,520)	S. pyogenes (*n* = 9,667)
PBP1a		
STMK^*a*^	445 (17.7)	0 (0)
SRN	0 (0)	0 (0)
KTG(T)	0 (0)	0 (0)
PBP2x		
STMK^*a*^	639 (25.3)	4 (0.04)
SSN	0 (0)	0 (0)
KSG(T)^*b*^	0 (3) [0 (0.1)]	0 (0)

a The transpeptidase domain sequences as defined in Li et al. (4) were truncated between the S and T of S/TMK.

b The two T553K sequences reported to be associated with β-lactam resistance in S. pyogenes in Vannice et al. (2) are not included.

10.1128/mSphere.00090-20.2 FIG S2 Streptococcus pneumoniae transpeptidase domain sequences mapped to the crystal structure of PBP2x. Shown is the crystal structure of PBP2X from S. pneumoniae bound to oxacillin (blue) with residue conservation of the 118 transpeptidase variants identified by Li et al. (4) mapped to the surface. The 118 nonredundant transpeptidase sequences identified by Li et al. (4) were aligned using MUSCLE aligner, the conservation of sites was mapped to the surface of PDB entry 5OIZ, and a color gradient was applied. Black residues represent regions absent from the alignment due to not being part of the transpeptidase domain. Thresholds were chosen to represent the range of sequence variation in the unique sequences but, unlike the S. pyogenes dataset, do not represent the frequency of the variants within the population. (Inset) Ribbon diagram of binding pocket motifs SSN, STMK, and KSG, with the position of the mutated residue (T550K) highlighted. Download FIG S2, PDF file, 1.0 MB. Copyright © 2020 Hayes et al. 2020 Hayes et al. This content is distributed under the terms of the Creative Commons Attribution 4.0 International license.

10.1128/mSphere.00090-20.6 TABLE S3 Differences in PBP2x protein sequence types compared to the consensus sequence. The associated numbers of variants from the most common sequence in both the full-length protein and transpeptidase domain, as well as the proportion in the population and amino acid changes, are shown. Red variants are in the same position (red boldface variants have the same amino acid change) as mutations associated with reduced susceptibility to β-lactams at subclinical concentrations (19). Download Table S3, DOCX file, 0.03 MB. Copyright © 2020 Hayes et al. 2020 Hayes et al. This content is distributed under the terms of the Creative Commons Attribution 4.0 International license.

**FIG 2:**
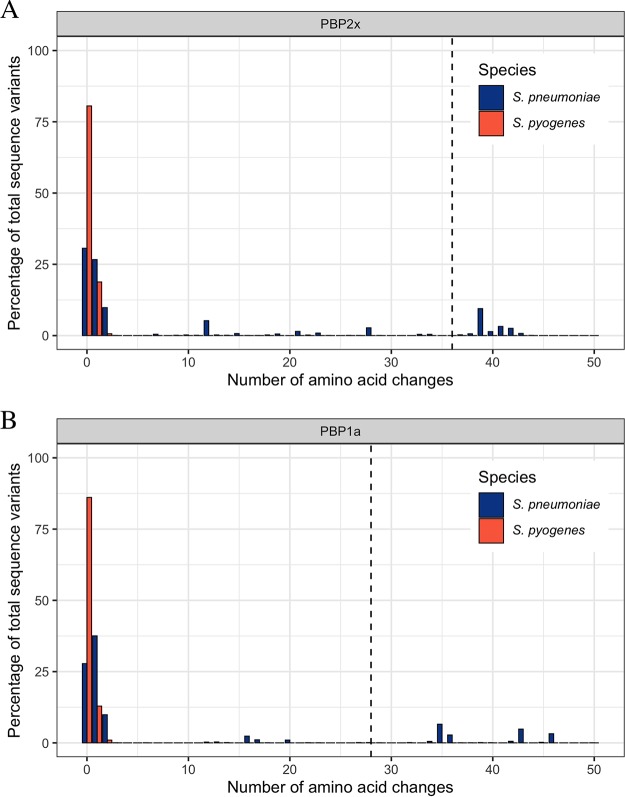
Amino acid differences of the transpeptidase domains of PBP2x and PBP1a. (A and B) The percentage of isolates with changes in the transpeptidase domains of PBP2x (A) and PBP1a (B) relative to penicillin-susceptible references in Streptococcus pneumoniae (blue; *n* = 2,520) and S. pyogenes (red; *n* = 9,667). Sequences that are >10% divergent (indicated by dotted vertical lines) have been associated with increased penicillin MICs in S. pneumoniae.

For S. pneumoniae, the number of substitutions across the whole transpeptidase domain of PBPs has been associated with penicillin resistance. Li et al. (4) found that penicillin MICs increased as the total number of divergent (defined as >10% amino acids different) transpeptidase domains of PBP2x, PBP1a, and PBP2b increased from 0 to 3. For S. pyogenes we used the most common amino acid sequences of PBP2x and PBP1a as our reference and, for S. pneumoniae, a previously defined wild type as the reference (4). There were considerably fewer PBP2x and PBP1a transpeptidase domains with multiple substitutions for S. pyogenes than for S. pneumoniae (Fig. 2). No S. pyogenes strains had sufficient mutations to reach the 10% threshold. For S. pneumoniae, 18.3% (462 of 2,520 strains) and 19.2% (485 of 2,520 strains) contained divergent PBP2x and PBP1a transpeptidase domains, respectively (Fig. 2). This pattern of greater conservation of S. pyogenes PBPs was also observed for PBP1b and PBP2a in S. pyogenes compared to PBP2b in S. pneumoniae (Fig. S3).

10.1128/mSphere.00090-20.3 FIG S3 Amino acid differences of the complete PBP1b and PBP2a for S. pyogenes and the transpeptidase domain of PBP2b for S. pneumoniae. The length of the complete PBP1b was 766 amino acids and PBP2a was 756 amino acids for S. pyogenes. The PBP2b transpeptidase domain was 280 amino acids in S. pneumoniae. Shown are the percentages of isolates with changes relative to penicillin-susceptible references in the full protein of S. pyogenes (red; *n*= 9,667) and in the transpeptidase domains of S. pneumoniae (blue; *n* = 2,520). (A) PBP1b (S. pyogenes); (B) PBP2a (S. pyogenes); (C) PBP2b (S. pneumoniae). Sequences that are >10% divergent are indicated by dotted vertical lines. Download FIG S3, PDF file, 0.2 MB. Copyright © 2020 Hayes et al. 2020 Hayes et al. This content is distributed under the terms of the Creative Commons Attribution 4.0 International license.

## DISCUSSION

Expanding on recent findings (19), we found no evidence that mutations are present in the β-lactam binding site KSGTAQ motif of PBP2x among 9,667 genetically and geographically diverse S. pyogenes genome sequences. While sporadic mutations were observed in PBP proteins, only four isolates contained mutations in the transpeptidase active sites of PBP2x and PBP1a. A further 84 strains (<1%) carried PBP2x amino acid variations recently associated with an increased tolerance to subclinical penicillin MIC (19). Although the report of two S. pyogenes isolates with clinical β-lactam resistance associated with *pbp2x* mutations is concerning (2), our findings provide reassurance that PBP mutations leading to clinical resistance are extremely limited, and perhaps unique, occurrences at this stage. Similar observations have been reported within closely related beta-hemolytic streptococci such as Streptococcus agalactiae and Streptococcus dysgalactiae subspecies *equisimilis*, where PBP mutations conferring reduced penicillin susceptibility or resistance were observed (18, 20), but without conclusive evidence of clonal expansion through current population-based surveillance investigations (6, 20).

We found a high degree of conservation of GAS PBP2x and PBP1a at transpeptidase active sites and across the broader transpeptidase domains. In comparison, PBP2x and PBP1a for S. pneumoniae were far less conserved, suggesting that there are strong evolutionary constraints in these domains for S. pyogenes that is not the case for S. pneumoniae. This may be due to several factors, including the lack of structural plasticity possible in PBP proteins of GAS (S. pyogenes lacks a PBP2b homolog), different β-lactam-resistant communities within the environmental niches occupied, lower natural transformation efficiency of GAS relative to that of S. pneumoniae, and a necessity for other chromosomal compensatory mutations to facilitate the maintenance of clinically relevant PBP mutations, as has been suggested for group B streptococci (2). Studies of penicillin-resistant S. pyogenes generated through mutagenesis (21) or serial passage in penicillin-containing medium (22) demonstrated that mutants with raised penicillin MICs appeared to have alterations in PBPs with reduced penicillin affinity (21). Notably, mutants grow more slowly, have aberrant colony morphology compared to that of wild-type strains (21), and are avirulent, with a decrease in M protein production (22). These laboratory experiments, together with the absence of naturally occurring isolates with greater than five amino acid substitutions in PBP2x or PBP1a, strongly suggest that changes to the PBPs are associated with a significant fitness cost. However, as subclinical low-level β-lactam resistance theoretically could confer biological advantages to S. pyogenes carriage, maintaining vigilance through population-based S. pyogenes surveillance for PBP variants is encouraged (19).

## MATERIALS AND METHODS

We obtained publicly available genome sequence data for 9,667 S. pyogenes isolates from the short-read archive (see Table S1 in the supplemental material). We assembled genomes using shovill v.1.0.9 (https://github.com/tseemann/shovill) with an underlying SKESA v.2.3.0 assembler (23). Using the β-lactam-susceptible S. pyogenes serotype M3 strain ATCC BAA-595/MGAS315 as a reference, we determined the presence, amino acid sequence, and alignment (24) of each of PBP2x, PBP1a, PBP1b, and PBP2a in each genome with the screen_assembly script (5) and BLASTP parameters of 100% coverage and 90% identity. Variant sites were identified from the multi-FASTA alignments using snp-sites (25).

To compare the conservation of the transpeptidase active-site motifs across streptococcal species, full-length PBP2x protein sequences of S. pyogenes serotype M3 strain ATCC BAA-595/MGAS315 (GenBank accession no. NC_004070.1), S. pneumoniae strain ATCC BAA-255/R6 (NC_003098.1), S. agalactiae strain 2603V/R (NC_004116.1), and the S. dysgalactiae subspecies *equisimilis* strain RE378 (NC_018712.1) reference genomes were aligned using Clustal Omega (26, 27). The percent sequence similarity was compared using Blosum62 with a threshold of 1 in Geneious Prime (28).

To investigate the inferred crystal structure location of S. pyogenes PBP2x mutations relative to that of the S. pneumoniae orthologue, S. pyogenes PBP2x sequence variations were plotted onto the S. pneumoniae PBP2x crystal structure bound to oxacillin (PDB entry 5OIZ) (29). Sequence conservation, as determined by the frequency (for S. pyogenes) and percentage (for S. pneumoniae) of variant amino acids compared to the consensus, was rendered onto the PBP2x crystal structure using UCSF Chimera (30).

We defined the PBP2x and PBP1a transpeptidase regions as those used in an assessment of 2,520 invasive S. pneumoniae isolates by Li et al. (4) and determined and plotted the number of pairwise amino acid differences within these regions using Distances Matrix in Geneious Prime (28) and ggplot2 in R version 3.6.1 (31). Similarly, we also assessed the conservation of PBP1b and PBP2a proteins for the 9,667 S. pyogenes genomes and the transpeptidase region of PBP2b for S. pneumoniae.

10.1128/mSphere.00090-20.7 Text S1 Sequences of 108 PBP1A amino acid variants identified within 9,667 global GAS genomes relative to the MGAS315 reference sequence SpyM3_1390. Download Text S1, TXT file, 0.2 MB. Copyright © 2020 Hayes et al. 2020 Hayes et al. This content is distributed under the terms of the Creative Commons Attribution 4.0 International license.

10.1128/mSphere.00090-20.8 Text S2 Sequences of 101 PBP1B amino acid variants identified within 9,667 global GAS genomes relative to the MGAS315 reference sequence SpyM3_0074. Download Text S2, TXT file, 0.2 MB. Copyright © 2020 Hayes et al. 2020 Hayes et al. This content is distributed under the terms of the Creative Commons Attribution 4.0 International license.

10.1128/mSphere.00090-20.9 Text S3 Sequences of 110 PBP2a amino acid variants identified within 9,667 global GAS genomes relative to the MGAS315 reference sequence SpyM3_1758. Download Text S3, TXT file, 0.2 MB. Copyright © 2020 Hayes et al. 2020 Hayes et al. This content is distributed under the terms of the Creative Commons Attribution 4.0 International license.

10.1128/mSphere.00090-20.10 Text S4 Sequences of 101 PBP2x amino acid variants identified within 9,667 global GAS genomes relative to the MGAS315 reference sequence SpyM3_1401. Download Text S4, TXT file, 0.2 MB. Copyright © 2020 Hayes et al. 2020 Hayes et al. This content is distributed under the terms of the Creative Commons Attribution 4.0 International license.

## References

[1] Horn DL, Zabriskie JB, Austrian R, Cleary PP, Ferretti JJ, Fischetti VA, Gotschlich E, Kaplan EL, McCarty M, Opal SM, Roberts RB, Tomasz A, Wachtfogel Y 1998 Why have group A streptococci remained susceptible to penicillin? Report on a symposium. Clin Infect Dis 26:1341–1345. doi:10.1086/516375. 9636860

[2] Vannice K, Ricaldi J, Nanduri S, Fang FC, Lynch J, Bryson-Cahn C, Wright T, Duchin J, Kay M, Chochua S, Van Beneden C, Beall B 2019 *Streptococcus pyogenes pbp2x* mutation confers reduced susceptibility to beta-lactam antibiotics. Clin Infect Dis 70:1265. doi:10.1093/cid/ciz1000. PMC7167332 31630171

[3] Dewe TCM, D’Aeth JC, Croucher NJ 2019 Genomic epidemiology of penicillin-non-susceptible *Streptococcus pneumoniae*. Microb Genom 5:e000305. doi:10.1099/mgen.0.000305. PMC6861860 31609685

[4] Li Y, Metcalf BJ, Chochua S, Li Z, Gertz RE Jr, Walker H, Hawkins PA, Tran T, Whitney CG, McGee L, Beall BW 2016 Penicillin-binding protein transpeptidase signatures for tracking and predicting beta-lactam resistance levels in *Streptococcus pneumoniae*. mBio 7:e00756-16. doi:10.1128/mBio.00756-16. 27302760 PMC4916381

[5] Davies MR, McIntyre L, Mutreja A, Lacey JA, Lees JA, Towers RJ, Duchene S, Smeesters PR, Frost HR, Price DJ, Holden MTG, David S, Giffard PM, Worthing KA, Seale AC, Berkley JA, Harris SR, Rivera-Hernandez T, Berking O, Cork AJ, Torres R, Lithgow T, Strugnell RA, Bergmann R, Nitsche-Schmitz P, Chhatwal GS, Bentley SD, Fraser JD, Moreland NJ, Carapetis JR, Steer AC, Parkhill J, Saul A, Williamson DA, Currie BJ, Tong SYC, Dougan G, Walker MJ 2019 Atlas of group A streptococcal vaccine candidates compiled using large-scale comparative genomics. Nat Genet 51:1035–1043. doi:10.1038/s41588-019-0417-8. 31133745 PMC6650292

[6] Athey TB, Teatero S, Sieswerda LE, Gubbay JB, Marchand-Austin A, Li A, Wasserscheid J, Dewar K, McGeer A, Williams D, Fittipaldi N 2016 High incidence of invasive group A *Streptococcus* disease caused by strains of uncommon *emm* types in Thunder Bay, Ontario, Canada. J Clin Microbiol 54:83–92. doi:10.1128/JCM.02201-15. 26491184 PMC4702752

[7] Ben Zakour NL, Davies MR, You Y, Chen JH, Forde BM, Stanton-Cook M, Yang R, Cui Y, Barnett TC, Venturini C, Ong CL, Tse H, Dougan G, Zhang J, Yuen KY, Beatson SA, Walker MJ 2015 Transfer of scarlet fever-associated elements into the group A *Streptococcus* M1T1 clone. Sci Rep 5:15877. doi:10.1038/srep15877. 26522788 PMC4629146

[8] Bergin SM, Periaswamy B, Barkham T, Chua HC, Mok YM, Fung DSS, Su AHC, Lee YL, Chua MLI, Ng PY, Soon WJW, Chu CW, Tan SL, Meehan M, Ang BSP, Leo YS, Holden MTG, De P, Hsu LY, Chen SL, de Sessions PF, Marimuthu K 2018 An outbreak of *Streptococcus pyogenes* in a mental health facility: advantage of well-timed whole-genome sequencing over *emm* typing. Infect Control Hosp Epidemiol 39:852–860. doi:10.1017/ice.2018.101. 29739475

[9] Chalker V, Jironkin A, Coelho J, Al-Shahib A, Platt S, Kapatai G, Daniel R, Dhami C, Laranjeira M, Chambers T, Guy R, Lamagni T, Harrison T, Chand M, Johnson AP, Underwood A, Scarlet Fever Incident Management Team. 2017 Genome analysis following a national increase in scarlet fever in England 2014. BMC Genomics 18:224. doi:10.1186/s12864-017-3603-z. 28283023 PMC5345146

[10] Chochua S, Metcalf BJ, Li Z, Rivers J, Mathis S, Jackson D, Gertz RE Jr, Srinivasan V, Lynfield R, Van Beneden C, McGee L, Beall B 2017 Population and whole genome sequence based characterization of invasive group A streptococci recovered in the United States during 2015. mBio 8:e01422-17. doi:10.1128/mBio.01422-17. 28928212 PMC5605940

[11] Coelho JM, Kapatai G, Jironkin A, Al-Shahib A, Daniel R, Dhami C, Laranjeira AM, Chambers T, Phillips S, Tewolde R, Underwood A, Chalker VJ 2019 Genomic sequence investigation *Streptococcus pyogenes* clusters in England (2010–2015). Clin Microbiol Infect 25:96–101. doi:10.1016/j.cmi.2018.04.011. 29698817

[12] Davies MR, Holden MT, Coupland P, Chen JH, Venturini C, Barnett TC, Zakour NL, Tse H, Dougan G, Yuen KY, Walker MJ 2015 Emergence of scarlet fever *Streptococcus pyogenes emm*12 clones in Hong Kong is associated with toxin acquisition and multidrug resistance. Nat Genet 47:84–87. doi:10.1038/ng.3147. 25401300

[13] Dickinson H, Reacher M, Nazareth B, Eagle H, Fowler D, Underwood A, Chand M, Chalker V, Coelho J, Daniel R, Kapatai G, Al-Shabib A, Puleston R 2019 Whole-genome sequencing in the investigation of recurrent invasive group A *Streptococcus* outbreaks in a maternity unit. J Hosp Infect 101:320–326. doi:10.1016/j.jhin.2018.03.018. 29577990

[14] Kapatai G, Coelho J, Platt S, Chalker VJ 2017 Whole genome sequencing of group A *Streptococcus*: development and evaluation of an automated pipeline for emm gene typing. PeerJ 5:e3226. doi:10.7717/peerj.3226. 28462035 PMC5410157

[15] Lynskey NN, Jauneikaite E, Li HK, Zhi X, Turner CE, Mosavie M, Pearson M, Asai M, Lobkowicz L, Chow JY, Parkhill J, Lamagni T, Chalker VJ, Sriskandan S 2019 Emergence of dominant toxigenic M1T1 *Streptococcus pyogenes* clone during increased scarlet fever activity in England: a population-based molecular epidemiological study. Lancet Infect Dis 19:1209–1218. doi:10.1016/S1473-3099(19)30446-3. 31519541 PMC6838661

[16] Turner CE, Bedford L, Brown NM, Judge K, Torok ME, Parkhill J, Peacock SJ 2017 Community outbreaks of group A *Streptococcus* revealed by genome sequencing. Sci Rep 7:8554. doi:10.1038/s41598-017-08914-x. 28819111 PMC5561225

[17] Dahesh S, Hensler ME, Van Sorge NM, Gertz RE Jr, Schrag S, Nizet V, Beall BW 2008 Point mutation in the group B streptococcal pbp2x gene conferring decreased susceptibility to beta-lactam antibiotics. Antimicrob Agents Chemother 52:2915–2918. doi:10.1128/AAC.00461-08. 18541727 PMC2493126

[18] Fuursted K, Stegger M, Hoffmann S, Lambertsen L, Andersen PS, Deleuran M, Thomsen MK 2016 Description and characterization of a penicillin-resistant *Streptococcus dysgalactiae* subsp. *equisimilis* clone isolated from blood in three epidemiologically linked patients. J Antimicrob Chemother 71:3376–3380. doi:10.1093/jac/dkw320. 27585966

[19] Musser JM, Beres SB, Zhu L, Olsen RJ, Vuopio J, Hyyrylainen HL, Grondahl-Yli-Hannuksela K, Kristinsson KG, Darenberg J, Henriques-Normark B, Hoffmann S, Caugant DA, Smith AJ, Lindsay DSJ, Boragine DM, Palzkill T 2020 Reduced in vitro susceptibility of *Streptococcus pyogenes* to beta-lactam antibiotics associated with mutations in the *pbp2x* gene is geographically widespread. J Clin Microbiol 58:e01993-19. doi:10.1128/JCM.01993-19. 31996443 PMC7098749

[20] Metcalf BJ, Chochua S, Gertz RE Jr, Hawkins PA, Ricaldi J, Li Z, Walker H, Tran T, Rivers J, Mathis S, Jackson D, Glennen A, Lynfield R, McGee L, Beall B, Active Bacterial Core Surveillance Team. 2017 Short-read whole genome sequencing for determination of antimicrobial resistance mechanisms and capsular serotypes of current invasive *Streptococcus agalactiae* recovered in the USA. Clin Microbiol Infect 23:574.e7–574.e14. doi:10.1016/j.cmi.2017.02.021. 28257899

[21] Gutmann L, Tomasz A 1982 Penicillin-resistant and penicillin-tolerant mutants of group A streptococci. Antimicrob Agents Chemother 22:128–136. doi:10.1128/aac.22.1.128. 6181734 PMC183685

[22] Rosendal K 1958 Investigations of penicillin-resistant streptococci belonging to group A. Acta Pathol Microbiol Scand 42:181–188. doi:10.1111/j.1699-0463.1958.tb03183.x. 13508261

[23] Souvorov A, Agarwala R, Lipman DJ 2018 SKESA: strategic k-mer extension for scrupulous assemblies. Genome Biol 19:153. doi:10.1186/s13059-018-1540-z. 30286803 PMC6172800

[24] Edgar RC 2004 MUSCLE: a multiple sequence alignment method with reduced time and space complexity. BMC Bioinformatics 5:113. doi:10.1186/1471-2105-5-113. 15318951 PMC517706

[25] Page AJ, Taylor B, Delaney AJ, Soares J, Seemann T, Keane JA, Harris SR 2016 SNP-sites: rapid efficient extraction of SNPs from multi-FASTA alignments. Microb Genom 2:e000056. doi:10.1099/mgen.0.000056. 28348851 PMC5320690

[26] Goujon M, McWilliam H, Li W, Valentin F, Squizzato S, Paern J, Lopez R 2010 A new bioinformatics analysis tools framework at EMBL-EBI. Nucleic Acids Res 38:W695–W699. doi:10.1093/nar/gkq313. 20439314 PMC2896090

[27] Sievers F, Wilm A, Dineen D, Gibson TJ, Karplus K, Li W, Lopez R, McWilliam H, Remmert M, Soding J, Thompson JD, Higgins DG 2011 Fast, scalable generation of high-quality protein multiple sequence alignments using Clustal Omega. Mol Syst Biol 7:539. doi:10.1038/msb.2011.75. 21988835 PMC3261699

[28] Kearse M, Moir R, Wilson A, Stones-Havas S, Cheung M, Sturrock S, Buxton S, Cooper A, Markowitz S, Duran C, Thierer T, Ashton B, Meintjes P, Drummond A 2012 Geneious Basic: an integrated and extendable desktop software platform for the organization and analysis of sequence data. Bioinformatics 28:1647–1649. doi:10.1093/bioinformatics/bts199. 22543367 PMC3371832

[29] Bernardo-Garcia N, Mahasenan KV, Batuecas MT, Lee M, Hesek D, Petrackova D, Doubravova L, Branny P, Mobashery S, Hermoso JA 2018 Allostery, recognition of nascent peptidoglycan, and cross-linking of the cell wall by the essential penicillin-binding protein 2x of *Streptococcus pneumoniae*. ACS Chem Biol 13:694–702. doi:10.1021/acschembio.7b00817. 29357220

[30] Pettersen EF, Goddard TD, Huang CC, Couch GS, Greenblatt DM, Meng EC, Ferrin TE 2004 UCSF Chimera–a visualization system for exploratory research and analysis. J Comput Chem 25:1605–1612. doi:10.1002/jcc.20084. 15264254

[31] R Core Team. 2019 R: a language and environment for statistical computing. R Foundation for Statistical Computing, Vienna, Austria https://www.R-project.org.

