# Supplementary material for: Restricted Sequence Variation in Streptococcus pyogenes Penicillin Binding Proteins
Source: mSphere. 2020 Apr 29;5(2):e00090-20. doi: 10.1128/mSphere.00090-20 (PMC7193039; doi:10.1128/mSphere.00090-20)
Supplement: FIG S1 [file mSphere.00090-20-sf001.pdf]

```
S.pneumoniae_R6_NP_357898.1            ----MKWTKRVIRYA--TKNRKSPAENRRRVGKSLSLLSVFVFAIFLVNFAVIIGTGTRF    54
S.agalactiae_2603V/R_NP_687322.1       MTFFKKLKKIFLDYVIHIRDRRSPQKNRERVGQNLMILTIFLFFIFIINFVIIVGTDSKF    60
S.pyogenes_MGAS315_WP_011106648.1      ---MKKWQKYVLDYV--VRDRRTPVENRVRVGQNMMLLTIFIFFIFIINFMIIIGTDQKF    55
S.equisimilis_RE378_WP_015017311.1     ---MRKWQKYFLDYV--VRDRRTPVQNRVRVGQNMMLLTIFVFFIFIINFMIIIGTDQKF    55
                                            *  * .: *.   ::*::* :** ***:.: :*::*:* **::** :*:**. :*

S.pneumoniae_R6_NP_357898.1            GTDLAKEAKKVHQTTRTVPAKRGTIYDRNGVPIAEDATSYNVYAVIDENYKSATGKILYV    114
S.agalactiae_2603V/R_NP_687322.1       GVNLSKEAKKVYQQSMTVQAKRGTIYDRNGNPIAEDATTYSLYAIISKNYTTATGQKLYV    120
S.pyogenes_MGAS315_WP_011106648.1      GVSLSEGAKKVYQETVTIQAKRGTIYDRNGTAIAVDSTTYSIYAILDKSFVSASDEKLYV    115
S.equisimilis_RE378_WP_015017311.1     GVSLSEGAKKVYQETVTVQAKRGTIYDRNGTAIAVDSTTYSIYAILDKSFVSASDEKLYV    115
                                       *..*:: ****:* : *: ***********  ** *:*:*.:**::.:.: :*:.: ***

S.pneumoniae_R6_NP_357898.1            EKTQFNKVAEVFHKYLDMEESYVREQLSQPNLKQVSFGAKGNGITYANMMSIKKELEAAE    174
S.agalactiae_2603V/R_NP_687322.1       QPSQYEKVASILENKLGMKKNLVLKQLNQKKLFQVSFGSSGSGLSYTKMADIKKTMEKSD    180
S.pyogenes_MGAS315_WP_011106648.1      QPSQYETVADILKKHLGMKKTDVIKQLKRKGLFQVSFGPSGSGISYSTMSTIQKAMEDAK    175
S.equisimilis_RE378_WP_015017311.1     QPSQYDKVAAILKEHLGMKKKDVIKQLKRKGLFQVSFGTSGSGISYSTMSTIQKAMEAAK    175
                                       : :*::.** ::.: *.*::. * :**.:  * ***** .*.*::*:.*  *:* :* :.

S.pneumoniae_R6_NP_357898.1            VKGIDFTTSPNRSYPNGQFASSFIGLAQLHENE-DGSKSLLGTSGMESSLNSILAGTDGI    233
S.agalactiae_2603V/R_NP_687322.1       IKGIGFSTSPGRIYPNGIFASQFIGFTLPQDDG--DGKKLVGNTGLEAALNKVLSGTDGK    238
S.pyogenes_MGAS315_WP_011106648.1      IKGIAFTTSPGRMYPNGTFASEFIGLASLTEDKKTGVKSLVGKTGLEASFDKILSGQDGV    235
S.equisimilis_RE378_WP_015017311.1     IKGIAFSASPGRMYPNGTFASEFIGLASLTEDKKTGVKSLVGKSGLEASFDKILSGQDGV    235
                                       :*** *::**.* **** ***.***::   ::   . *.*:*.:*:*::::.:*:* **

S.pneumoniae_R6_NP_357898.1            ITYEKDRLGNIVPGTEQVSQRTMDGKDVYTTISSPLQSFMETQMDAFQEKVKGKYMTATL    293
S.agalactiae_2603V/R_NP_687322.1       VTYEKDRSGNVLLGTATTERRAVNGKDIYTTLSEPIQTVLETQMDVFAEKTKGKFASATV    298
S.pyogenes_MGAS315_WP_011106648.1      ITYQKDRNGATLLGTGKTVKKAIDGKDIYTTLSEPIQTFLETQMDVFQAKSNGQLASATL    295
S.equisimilis_RE378_WP_015017311.1     ITYQKDRNGNTLLGTGKTVKKAVDGKDIYTTLSEPIQTFLETQMDIFQAKSNGKLATATL    295
                                       :**:*** *  :  ** . ::::***:***:*.*:*:.:*****  *  * :*:  :**:

S.pneumoniae_R6_NP_357898.1            VSAKTGEILATTQRPTFDADTKEGITE-DFVW-RDILYQSNYEPGSTMKVMMLAAAIDNN    351
S.agalactiae_2603V/R_NP_687322.1       VNAKTGEILATSQRPTYNPSTLKGYDKKNLGTYNTLLYDNFFEPGSTMKVMTLASAIDSK    358
S.pyogenes_MGAS315_WP_011106648.1      VNAKTGEILATTQRPTYNADTLKGLENTNYKWYSALH-QGNFEPGSTMKVMTLAAAIDDK    354
S.equisimilis_RE378_WP_015017311.1     VNAKTGEILATTQRPTYNADTLKGLENKDYKWYSALH-QGNFEPGSTMKVMTLAAAIDDK    354
                                       *.*********:****:: .* :*  : :      :  :. :********* **:***.:

S.pneumoniae_R6_NP_357898.1            TFPGGEVFNS-SELKIADATIRDWDVNEGLTGGRMMTFSQGFAHSSNVGMTLLEQKMGDA    410
S.agalactiae_2603V/R_NP_687322.1       HFNSTEVYNS-AQYKIADAIIRDWDVNEGLSSGSYMTFPQGFAHSSNVGMVTLEQKMGRD    417
S.pyogenes_MGAS315_WP_011106648.1      VFNPNETFSNANGLTIADATIQDWSINEGISTGQYMNYAQGFAFSSNVGMTKLEQKMGNA    414
S.equisimilis_RE378_WP_015017311.1     VFNPNETFSNANGLTIADATIQDWAINEGISTGQYMNYAQGFAFSSNVGMTKLEQKMGNA    414
                                        *   *.:..    .**** *:** :***:: *  *.: ****.******. ******
```

```
S.pneumoniae_R6_NP_357898.1           TWLDYLNRFKFGVPTRFGLTDEYAGQLPADNIVNIAQSSFGQGISVTQTQMIRAFTAIAN   470
S.agalactiae_2603V/R_NP_687322.1      KWLNYLSKFKFGYPTRFGMLHESGGLFPSDNEVTIAMSSFGQGIGVTQVQMLRAFTSISN   477
S.pyogenes_MGAS315_WP_011106648.1     KWMNYLTKFRFGFPTRFGLKDEDAGIFPSDNIVTQAMSAFGQGISVTQIQMLRAFTAISN   474
S.equisimilis_RE378_WP_015017311.1    KWMNYLTKFRFGFPTRFGLQDEYAGMLPSDNIVTQAMSAFGQGISVTKAQMLRAFTAISN   474
                                      .*::**.:*:** *****: .* .* :*:** *. * *:*****.**: **:****:*:*

S.pneumoniae_R6_NP_357898.1           DGVMLEPKFISAIYDPNDQTARKSQKEIVGNPVSKDAASLTRTNMVLVGTDPVYGTMYNH   530
S.agalactiae_2603V/R_NP_687322.1      DGVMLQPQFISSIYDPNTGTSRTARKEVVGKPVSKEAASKTRDYMVTVGTDPYYGTLYAA   537
S.pyogenes_MGAS315_WP_011106648.1     NGEMLEPQFISQIYDPNTASFRTANKEIVGKPVSKKAASETRQYMIGVGTDPEFGTLYSK   534
S.equisimilis_RE378_WP_015017311.1    DGEMLEPQFVSQIYDPNTASFRTAQKEVVGKPVSKKAASDTRHYMIGVGTDPEFGTLYSK   534
                                      :* **:*:*:* *****  : *.:.**:**:****.*** **  *: ***** :**:*

S.pneumoniae_R6_NP_357898.1           STGKPTVTVPGQNVALKSGTAQIADEKNGGYLVG-LTDYIFSAVSMSPAENPDFILYVTV   589
S.agalactiae_2603V/R_NP_687322.1      G--APVIQVGNQSVAVKSGTAQIAQEGGGGYLQGK-NDTINSVVAMVPSENPDFIMYVTI   594
S.pyogenes_MGAS315_WP_011106648.1     TF-GPIIKVGDLPVAVKSGTAQIGSEDGSGYQDGGLTNYVYSVVAMVPADKPDFLMYVTM   593
S.equisimilis_RE378_WP_015017311.1    TY-GPIIKVGDLPVAVKSGTAQIGAEDGSGYQDGGLTNYVYSVVAMVPADKPDFVMYVTM   593
                                          * : * .  **:*******. * ..**  *  .: : *.*:* *:::***::***:

S.pneumoniae_R6_NP_357898.1           QQPEHYSGIQLGEFANPILERASAMKDSLNLQTTAKALEQVSQQSPYPMPSVKDISPGDL   649
S.agalactiae_2603V/R_NP_687322.1      QQPEKFSITFWKDVVNPVLEQATAMKETILKPG----LNDSEHQTKYKLSKIVGENPGHV   650
S.pyogenes_MGAS315_WP_011106648.1     TKPQHFGPLFWQDVVNPVLEEAYLMQDTLTKPV----VSDANRQTTYKLPNFVGKNPGET   649
S.equisimilis_RE378_WP_015017311.1    TQPEHFGPLFWQDVVNPVLEEAYLMQDTLTRPV----AADDAHKTTYQLPDFIGKNPGET   649
                                       :*:::.     :..**:**.*  *::::          :  ::: * : .. . .**.

S.pneumoniae_R6_NP_357898.1           AEELRRNLVQPIVVGTGTKIKNSSAEEGKNLAPNQQVLILSDKAEEVPDMYGWTKETAET   709
S.agalactiae_2603V/R_NP_687322.1      AEELRRNLVQPIILGNGSKVSKVSKRPGANLAENEQLLVLTNKLTELPDMYGWSKANVEQ   710
S.pyogenes_MGAS315_WP_011106648.1     SSELRRNLVQPVVLGTGSKIKKVSHQPGQTLTENQQVLILSDRFVEVPDMYGWTKSNVKT   709
S.equisimilis_RE378_WP_015017311.1    SSELRRNLVQPVVLGTGSKIKKISQKAGKGLSENQQILILSDHFTELPDMYGWTKSNVKT   709
                                      :.*********:::*.*:*:.: * . *  *: *:*:*:*:::  *:******:* ..:

S.pneumoniae_R6_NP_357898.1           LAKWLNIELEFQGSGS-TVQKQDVRANTAIKDIKKITLTLGD----   750
S.agalactiae_2603V/R_NP_687322.1      FAKWTGIKVTYKGSTSGKVRKQSIDVGKSINKIKKIKITIGD----   752
S.pyogenes_MGAS315_WP_011106648.1     FAKWTGIDISFKGTDSGRVMKQSVDVGKSLKKIKKMTITLGD----   751
S.equisimilis_RE378_WP_015017311.1    FAKWTGIDVTFKGSDSGRVTKQSLDVGKSLEKNQKNYHNFRRLTCF   755
                                      :*** .*.: ::*: *  * **.: ...:::. :*   .:
```
