# Supplementary material for: Restricted Sequence Variation in Streptococcus pyogenes Penicillin Binding Proteins
Source: mSphere. 2020 Apr 29;5(2):e00090-20. doi: 10.1128/mSphere.00090-20 (PMC7193039; doi:10.1128/mSphere.00090-20)
Supplement: TABLE S2 [file mSphere.00090-20-st002.docx]

|  | *S. pneumoniae* | *S. agalactiae* | *S. pyogenes* | *S. equisimilis* |
| --- | --- | --- | --- | --- |
| *S. pneumoniae*_R6_NP_357898.1 |  | 72.619 | 73.413 | 72.354 |
| *S. agalactiae*_2603V/R_NP_687322.1 | 72.619 |  | 79.31 | 78.647 |
| *S. pyogenes*_MGAS315_WP_011106648.1 | 73.413 | 79.31 |  | 95.739 |
| *S. equisimilis*_RE378_WP_015017311.1 | 72.354 | 78.647 | 95.739 |  |
