# Supplementary figures and images for: Restricted Sequence Variation in Streptococcus pyogenes Penicillin Binding Proteins
Source: mSphere. 2020 Apr 29;5(2):e00090-20. doi: 10.1128/mSphere.00090-20 (PMC7193039; doi:10.1128/mSphere.00090-20)

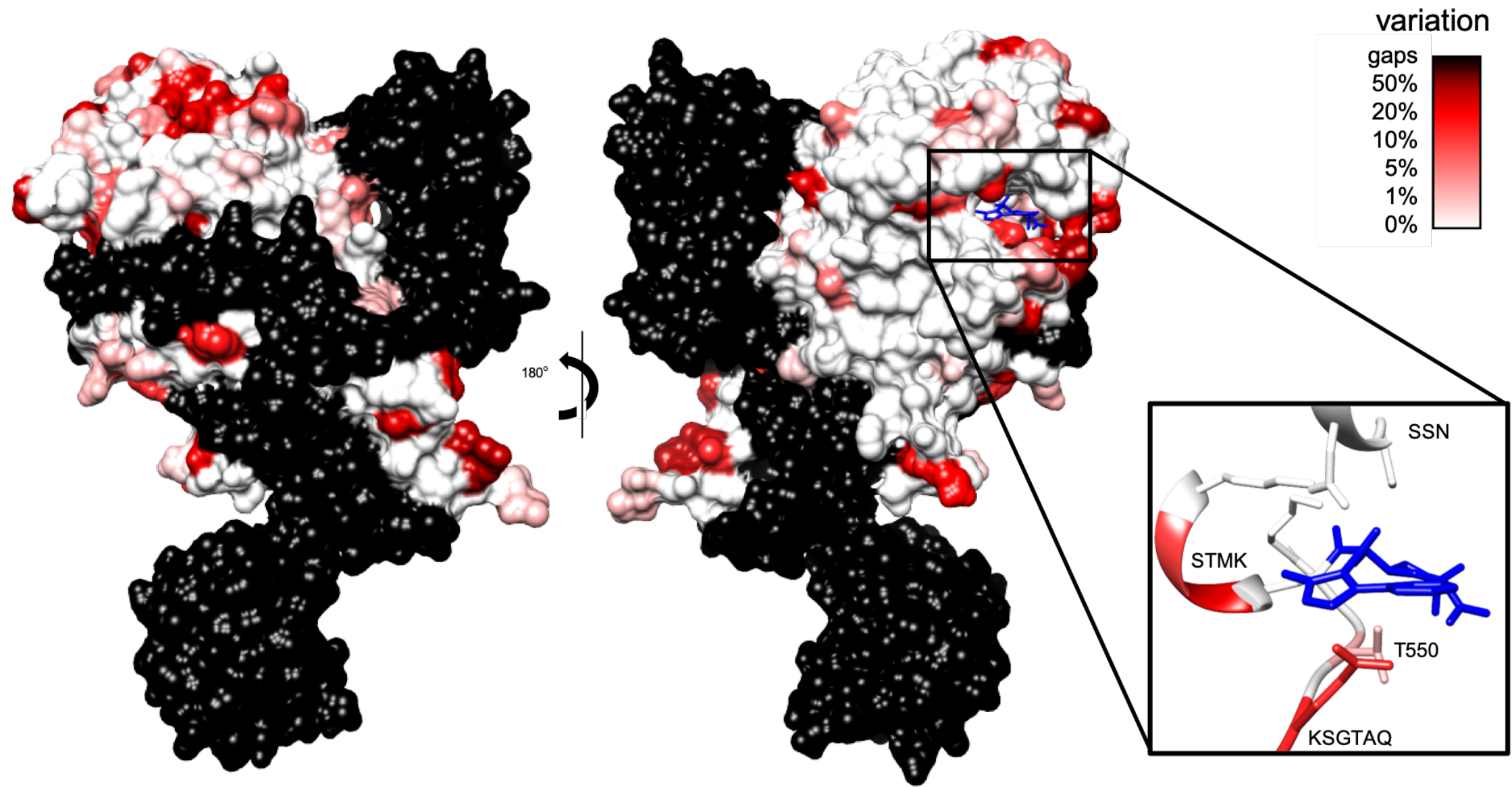

Supplement: FIG S2 [file mSphere.00090-20-sf002.pdf]

A

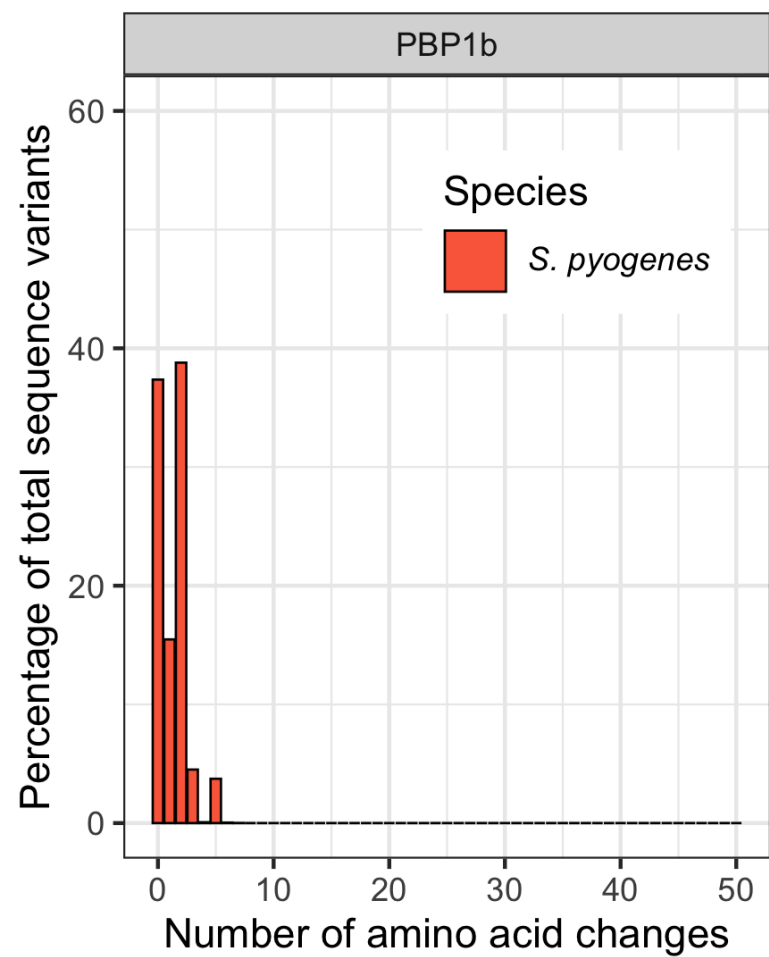

B

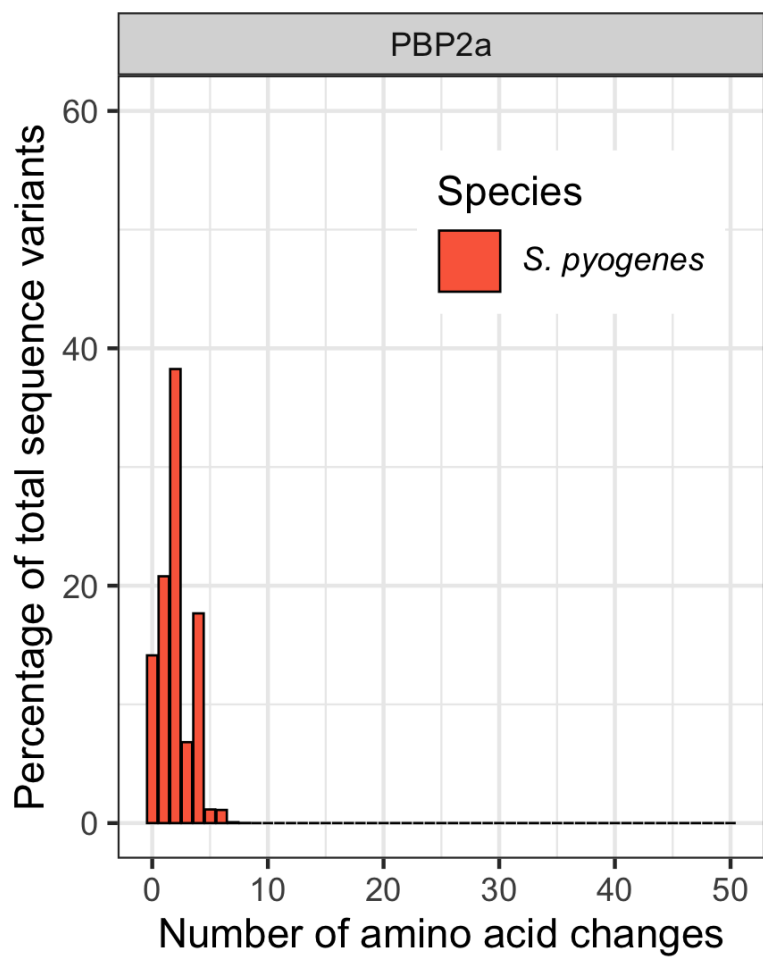

C

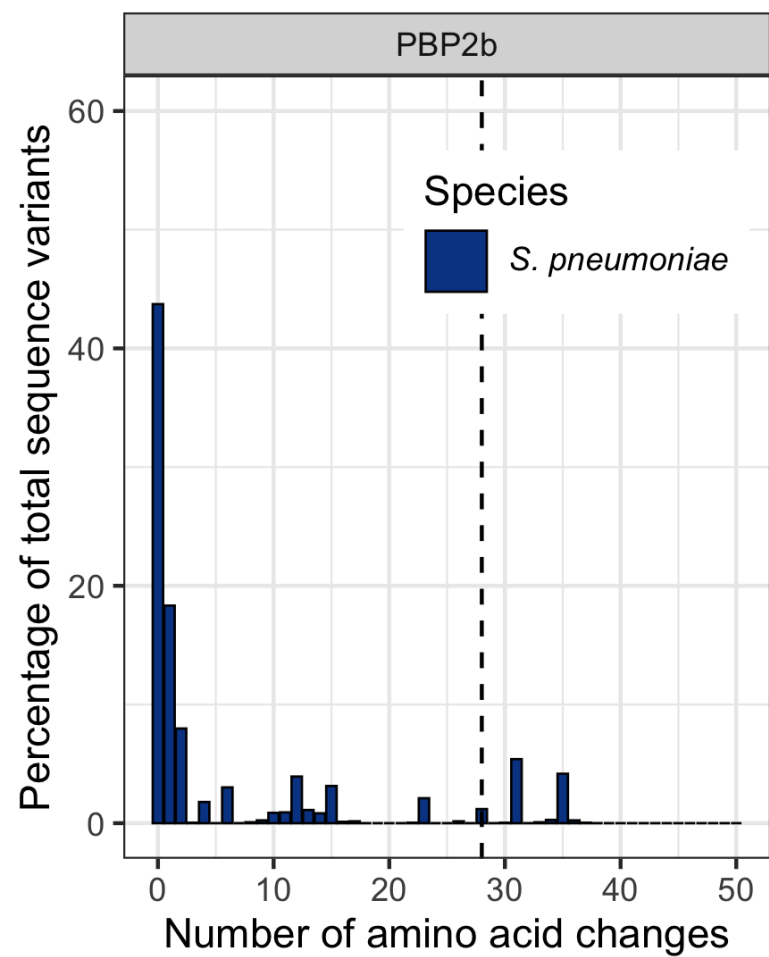

Supplement: FIG S3 [file mSphere.00090-20-sf003.pdf]
