# Supplementary material for: Restricted Sequence Variation in Streptococcus pyogenes Penicillin Binding Proteins
Source: mSphere. 2020 Apr 29;5(2):e00090-20. doi: 10.1128/mSphere.00090-20 (PMC7193039; doi:10.1128/mSphere.00090-20)
Supplement: TABLE S3 [file mSphere.00090-20-st003.docx]

| **PBP2x type** | **No. variants (full sequence)** | **No. variants transpeptidase (291-603)** | **No. of isolates** | **% population**  **(n=9,667)** | **Variants** | | | |
| --- | --- | --- | --- | --- | --- | --- | --- | --- |
| **1(MGAS315)** | **2** | **0** | **1085** | 11.19 | T245A |  |  |  |
| **2** | **4** | **1** | **468** | 4.83 | I502V | P676S | K708E |  |
| **3** | **1** | **0** | **5983** | 61.70 |  |  |  |  |
| **4** | **3** | **2** | **15** | 0.15 | G600A | P601H |  |  |
| **5** | **2** | **1** | **82** | 0.85 | M593T |  |  |  |
| **6** | **2** | **1** | **1015** | 10.47 | S562T |  |  |  |
| **7** | **3** | **1** | **34** | 0.35 | S562T | P676S | K708E |  |
| **8** | **4** | **1** | **13** | 0.13 | I35T | I502V | P676S | K708E |
| **9** | **2** | **0** | **45** | 0.46 | K213N | T246A |  |  |
| **10** | **1** | **0** | **4** | 0.04 | R632H |  |  |  |
| **11** | **1** | **0** | **2** | 0.02 | E269D |  |  |  |
| **12** | **1** | **0** | **31** | 0.32 | A108T |  |  |  |
| **13** | **1** | **0** | **5** | 0.05 | D173G |  |  |  |
| **14** | **1** | **1** | **2** | 0.02 | P526S |  |  |  |
| **15** | **2** | **0** | **2** | 0.02 | I47V | T245A |  |  |
| **16** | **1** | **0** | **161** | 1.66 | D125Y |  |  |  |
| **17** | **1** | **1** | **1** | 0.01 | D544N |  |  |  |
| **18** | **1** | **0** | **15** | 0.15 | I48V |  |  |  |
| **19** | **1** | **1** | **10** | 0.10 | **P601L** |  |  |  |
| **20** | **1** | **0** | **2** | 0.02 | M46I |  |  |  |
| **21** | **2** | **1** | **3** | 0.03 | I97V | S562T |  |  |
| **22** | **2** | **0** | **4** | 0.04 | T245A | T635A |  |  |
| **23** | **1** | **0** | **30** | 0.31 | D226N |  |  |  |
| **24** | **4** | **1** | **1** | 0.01 | I502V | P676S | K708E | M729I |
| **25** | **1** | **0** | **244** | 2.52 | I73M |  |  |  |
| **26** | **1** | **1** | **3** | 0.03 | D353A |  |  |  |
| **27** | **1** | **0** | **11** | 0.11 | G701S |  |  |  |
| **28** | **1** | **0** | **1** | 0.01 | I139T |  |  |  |
| **29** | **2** | **1** | **3** | 0.03 | T245A | G521S |  |  |
| **30** | **1** | **0** | **1** | 0.01 | V235I |  |  |  |
| **31** | **1** | **0** | **3** | 0.03 | T136I |  |  |  |
| **32** | **1** | **1** | **45** | 0.46 | G600D |  |  |  |
| **33** | **2** | **0** | **10** | 0.10 | P676S | K708E |  |  |
| **34** | **2** | **2** | **1** | 0.01 | A393T | S562T |  |  |
| **35** | **2** | **2** | **1** | 0.01 | T341A | S562T |  |  |
| **36** | **1** | **1** | **1** | 0.01 | T307A |  |  |  |
| **37** | **1** | **1** | **1** | 0.01 | V503I |  |  |  |
| **38** | **1** | **1** | **1** | 0.01 | A330T |  |  |  |
| **39** | **1** | **0** | **1** | 0.01 | I42F |  |  |  |
| **40** | **2** | **2** | **19** | 0.20 | A397V | I502V |  |  |
| **41** | **4** | **2** | **11** | 0.11 | T294A | D353A | P676S | K708E |
| **42** | **1** | **0** | **1** | 0.01 | E61G |  |  |  |
| **43** | **1** | **0** | **1** | 0.01 | V696I |  |  |  |
| **44** | **1** | **0** | **1** | 0.01 | D52G |  |  |  |
| **45** | **1** | **0** | **1** | 0.01 | V735I |  |  |  |
| **46** | **2** | **1** | **1** | 0.01 | T535I | S738P |  |  |
| **47** | **2** | **2** | **1** | 0.01 | **M342I** | S562T |  |  |
| **48** | **2** | **1** | **1** | 0.01 | S562T | T703I |  |  |
| **49** | **2** | **1** | **1** | 0.01 | S562T | S725F |  |  |
| **50** | **3** | **1** | **1** | 0.01 | D125N | I502V | P676S |  |
| **51** | **1** | **1** | **3** | 0.03 | I502V |  |  |  |
| **52** | **4** | **0** | **1** | 0.01 | S104T | T245A | P676S | K708E |
| **53** | **3** | **1** | **79** | 0.81 | D353A | P676S | K708E |  |
| **54** | **1** | **1** | **1** | 0.01 | A493T |  |  |  |
| **55** | **1** | **0** | **1** | 0.01 | D734G |  |  |  |
| **56** | **1** | **0** | **1** | 0.01 | M46T |  |  |  |
| **57** | **1** | **0** | **1** | 0.01 | T277A |  |  |  |
| **58** | **1** | **1** | **9** | 0.09 | M593V |  |  |  |
| **59** | **1** | **0** | **8** | 0.08 | T649I |  |  |  |
| **60** | **1** | **0** | **7** | 0.07 | V57F |  |  |  |
| **61** | **2** | **2** | **8** | 0.08 | S562T | **P601L** |  |  |
| **62** | **1** | **1** | **4** | 0.04 | A291T |  |  |  |
| **63** | **2** | **0** | **1** | 0.01 | V57F | I742V |  |  |
| **64** | **4** | **1** | **1** | 0.01 | I502V | P676S | K708E | V733I |
| **65** | **2** | **1** | **3** | 0.03 | D233Y | S562T |  |  |
| **66** | **1** | **0** | **2** | 0.02 | D207G |  |  |  |
| **67** | **3** | **1** | **1** | 0.01 | G62E | I502V | P676S |  |
| **68** | **1** | **1** | **1** | 0.01 | I458V |  |  |  |
| **69** | **1** | **0** | **1** | 0.01 | V212A |  |  |  |
| **70** | **2** | **2** | **1** | 0.01 | M593V | **P601L** |  |  |
| **71** | **2** | **0** | **1** | 0.01 | T665I | S732N |  |  |
| **72** | **1** | **0** | **1** | 0.01 | T703S |  |  |  |
| **73** | **1** | **0** | **100** | 1.03 | V626I |  |  |  |
| **74** | **1** | **1** | **2** | 0.02 | P601H |  |  |  |
| **75** | **2** | **1** | **2** | 0.02 | I228T | S562T |  |  |
| **76** | **1** | **1** | **15** | 0.15 | A397V |  |  |  |
| **77** | **1** | **1** | **1** | 0.01 | T307I |  |  |  |
| **78** | **1** | **1** | **1** | 0.01 | M593L |  |  |  |
| **79** | **1** | **0** | **1** | 0.01 | A284V |  |  |  |
| **80** | **2** | **2** | **1** | 0.01 | S562T | P601H |  |  |
| **81** | **1** | **1** | **1** | 0.01 | I463L |  |  |  |
| **82** | **1** | **0** | **1** | 0.01 | A108V |  |  |  |
| **83** | **4** | **2** | **1** | 0.01 | N357H | I502V | P676S | K708E |
| **84** | **2** | **2** | **2** | 0.02 | T369K | S562T |  |  |
| **85** | **1** | **1** | **1** | 0.01 | **M342I** |  |  |  |
| **86** | **1** | **0** | **1** | 0.01 | M188L |  |  |  |
| **87** | **1** | **0** | **2** | 0.02 | K741Q |  |  |  |
| **88** | **2** | **2** | **1** | 0.01 | S562T | L602F |  |  |
| **89** | **1** | **0** | **5** | 0.05 | S732N |  |  |  |
| **90** | **1** | **0** | **1** | 0.01 | P676T |  |  |  |
| **91** | **1** | **0** | **1** | 0.01 | D734E |  |  |  |
| **92** | **1** | **1** | **1** | 0.01 | T346A |  |  |  |
| **93** | **1** | **0** | **1** | 0.01 | V642A |  |  |  |
| **94** | **1** | **1** | **1** | 0.01 | I540L |  |  |  |
| **95** | **1** | **0** | **2** | 0.02 | E206V |  |  |  |
| **96** | **1** | **0** | **1** | 0.01 | T277N |  |  |  |
| **97** | **2** | **1** | **1** | 0.01 | S562T | E614K |  |  |
| **98** | **2** | **1** | **1** | 0.01 | S562T | G643R |  |  |
| **99** | **1** | **1** | **2** | 0.02 | F356L |  |  |  |
| **100** | **1** | **0** | **1** | 0.01 | P625S |  |  |  |
| **101** | **1** | **0** | **1** | 0.01 | T709I |  |  |  |
